# LY2157299 Monohydrate, a TGF-βR1 Inhibitor, Suppresses Tumor Growth and Ascites Development in Ovarian Cancer

**DOI:** 10.3390/cancers10080260

**Published:** 2018-08-07

**Authors:** Qing Zhang, Xiaonan Hou, Bradley J. Evans, Jamison L. VanBlaricom, Saravut J. Weroha, William A. Cliby

**Affiliations:** 1 Department of Obstetrics and Gynecology, Mayo Clinic, Rochester, MN 55905, USA; zhang.qing@mayo.edu; 2 Department of Oncology, Mayo Clinic, Rochester, MN 55905, USA; hou.xiaonan@mayo.edu (X.H.); Evans.Bradley@mayo.edu (B.J.E.); VanBlaricom.Jamison@mayo.edu (J.L.V.); Weroha.Saravut@mayo.edu (S.J.W.)

**Keywords:** high-grade serous ovarian cancer, TGF-β signaling, LY2157299 monohydrate, patient-derived xenografts, stroma, ascites

## Abstract

Transforming growth factor beta (TGF-β) signaling has pleiotropic functions regulating cancer initiation, development, and metastasis, and also plays important roles in the interaction between stromal and cancer cells, making the pathway a potential therapeutic target. LY2157299 monohydrate (LY), an inhibitor of TGF-β receptor I (TGFBRI), was examined for its ability to inhibit ovarian cancer (OC) growth both in high-grade serous ovarian cancer (HGSOC) cell lines and xenograft models. Immunohistochemistry, qRT-PCR, and Western blot were performed to study the effect of LY treatment on expression of cancer- and fibroblast-derived genes. Results showed that exposure to TGF-β1 induced phosphorylation of SMAD2 and SMAD3 in all tested OC cell lines, but this induction was suppressed by pretreatment with LY. LY alone inhibited the proliferation, migration, and invasion of HGSOC cells in vitro. TGF-β1-induced fibroblast activation was blocked by LY. LY also delayed tumor growth and suppressed ascites formation in vivo. In addition, independent of tumor inhibition, LY reduces ascites formation in vivo. Using OVCAR8 xenograft specimens we confirmed the inhibitory effect of LY on TGF-β signaling and tumor stromal expression of collagen type XI chain 1 (COL11A1) and versican (VCAN). These observations suggest a role for anti-TGF-β signaling-directed therapy in ovarian cancer.

## 1. Introduction

Ovarian cancer (OC) is the most lethal gynecologic cancer, with an estimated 238,700 new cases annually worldwide [1]. In the United States alone there will be an estimated 14,070 deaths during 2018 [2]. The standard treatment for OC is primary surgical cytoreduction followed by adjuvant chemotherapy. Although 70% of the patients respond well to the initial adjuvant chemotherapy, most of these patients relapse and evolve to a chemoresistant phenotype. Attempts to delay the onset of recurrence or chemoresistance following first-line therapy by use of maintenance therapy have only shown modest improvements [3,4,5,6,7,8].

In OC, cancer-associated stroma stimulates proliferation, migration, and invasion and these effects are at least partially modulated by transforming growth factor beta (TGF-β) signaling [9,10]. TGF-β has dual roles in cancer and has been shown to act as both a tumor suppressor and promoter. Relevant to the present study, at the level of the tumor microenvironment, TGF-β signaling promotes a favorable microenvironment for tumor implantation, growth, and therefore evolution of metastasis [11,12,13,14]. In this setting, TGF-β signaling regulates the cancer–stromal interaction to stimulate tumor growth [9,10], and inhibits T cell- and natural killer (NK) cell-mediated tumor clearance to escape from host immunosurveillance [13,15,16,17]. Many TGF-β signaling-related genes have been reported to play roles in cancer–stromal crosstalk, and these genes are more likely derived from cancer-associated fibroblasts (CAFs) rather than cancer cells, e.g., collagen type V alpha 1 chain (COL5A1), collagen type XI chain 1 (COL11A1), tissue inhibitor of metalloproteinases 3 (TIMP3), and versican (VCAN) [9,10,18]. Blockade of TGF-β signaling has been shown to inhibit tumor progression and metastasis in cellular and animal studies in a wide variety of human cancers [12,19,20,21,22,23]. Calon et al. reported that the TGFBRI inhibitor LY2157299 monohydrate (galunisertib, LY), inhibited tumor initiation in colorectal cancer xenograft models [24]. Alsina-Sanchis et al. treated OC patient-derived xenografts (PDXs) with a TGF-β receptor I and II dual inhibitor, LY2109761 and observed a reduction in tumor size [25]. Consequently, several different TGF-β inhibitors have been developed for potential anticancer treatments [26].

Among these inhibitors, LY2157299 monohydrate is a small molecular inhibitor of TGFBRI that has demonstrated less toxicity and stronger activity against pancreatic and lung cancer in phase I clinical trials. It is currently being evaluated in phase II trials [20,26,27,28]. However, prior studies using LY alone in cell culture or xenograft models displayed limited anti-tumor activity [19,23]. Calon et al. demonstrated that LY inhibited colorectal tumor metastases through targeting TGF-β signaling in the tumor microenvironment [24]. This study provides evidence that LY may function by targeting the cancer-associated stroma rather than cancer cell itself. In the present manuscript we tested the ability of LY to inhibit the growth and/or engraftment in OC in vivo. 

## 2. Results

### 2.1. TGFB1 and TGFBR1 Are Expressed, and TGF-β Signaling Is Active Across OC Cell Lines and PDXs 

**Figure 1:**
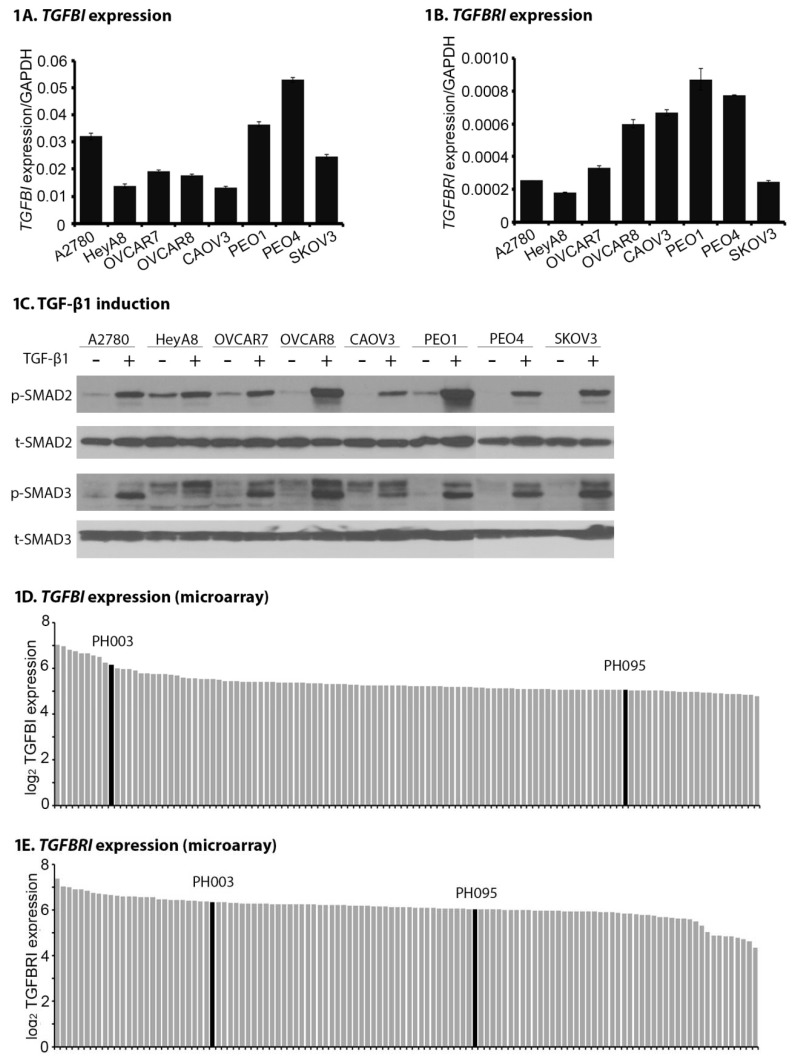
Ovarian cancer (OC) cell lines and patient-derived xenografts (PDXs) express the transforming growth factor beta (TGF-β) ligand and receptor and demonstrate intact SMAD signaling. The expression of *TGFBI* (**A**) and *TGFBRI* (**B**) were measured by qPCR across eight OC cell lines (A2780, OV202, PEO1, OVCAR7, OVCAR8, SKOV3, HeyA8, and CAOV3). *GAPDH* was used as an internal control. Data represent the mean ± standard deviation (SD) of three independent experiments. (**C**) Western blot analysis of p-SMAD2 and p-SMAD3 following TGF-β1 (5 ng/mL) treatment for 1 h in OC cell lines. Total SMAD2 and SMAD3 were used as loading controls. Microarray analysis of expression of *TGFBI* (**D**) and *TGFBRI* (**E**) in a cohort of OC PDXs (*n* = 118). PH003 and PH095 were selected for the following in vivo testing (denoted by black bars).

Real-time qPCR was performed to evaluate the mRNA expression of two TGF-β signaling pathway-related genes, *TGFBI* and *TGFBRI*, across eight OC cell lines. As shown in Figure 1, both *TGFBI* (Figure 1A) and *TGFBRI* (Figure 1B) show relative expression levels across eight OC cell lines. There was a narrow range of transcript levels from (13.3 ± 0.3) × 10^−3^ (CAOV3) to (53.0 ± 0.9) × 10^−3^ (PEO4) for *TGFBI*; and (1.8 ± 0.1) × 10^−4^ (HeyA8) to (8.7 ± 1.2) × 10^−4^ (PEO1) for *TGFBRI*, relative to the *GAPDH* internal control. We confirmed that all tested cell lines are capable of inducing phosphorylation of SMAD2 (p-SMAD2) and phosphorylation of SMAD3 (p-SMAD3) in response to TGF-β1 stimulation (Figure 1C), suggesting intact TGF-β signaling.

We have previously reported that OC PDXs show pathologic, molecular, and treatment-responsive similarities to the source patients [29]. Further microarray analysis on 118 PDX models was performed to assess TGF-β signaling pathway-related genes in xenograft models. Results show that the expression of *TGFBI* (Figure 1D) and *TGFBRI* (Figure 1E) is relatively consistent across PDX models, which is consistent with the qRT-PCR and Western blot results in OC cell lines (Figure 1A–C). Collectively, these data demonstrate that TGF-β signaling pathway is functional in both OC cells and PDX models.

### 2.2. LY Suppresses Proliferation, Migration, and Invasion of OC Cells In Vitro

LY can inhibit cell growth and migration in hepatocellular carcinoma, pancreatic cancer, and glioma cells [21,30,31]. However, the efficacy of LY in OC cells has not been tested. In this study, two HGSOC cell lines, OVCAR8 and CAOV3 [32], were chosen to study the effects of this inhibitor. 

**Figure 2:**
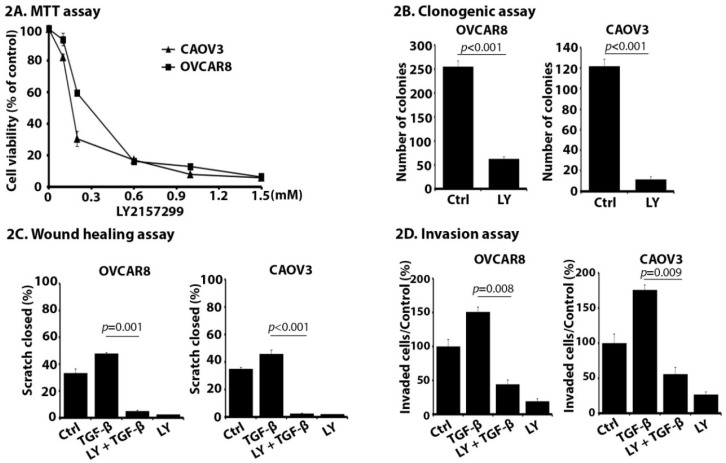
LY2157299 monohydrate (LY) suppresses proliferation, migration, and invasion in high-grade serous ovarian cancer (HGSOC) cell lines in vitro. OVCAR8 and CAOV3 cell lines were treated with LY with or without TGF-β1 followed by (**A**) MTT cell proliferation assay, (**B**) Clonogenic assay, (**C**) Wound-healing assay, and (**D**) Matrigel invasion assay. All data represent the mean ± SD of three independent experiments.

MTT assay showed that LY exhibited cytotoxicity in OC cell lines with IC50 values (mean ± SD) of 226.71 ± 4.87 μM and 159.93 ± 3.94 μM for OVCAR8 and CAOV3, respectively (Figure 2A). The long-term effect of LY on OC cell proliferation was also studied by clonogenic assay, showing that LY could significantly inhibit OC cell colony formation at IC25 of LY (Figure 2B, *p* < 0.001 for both cell lines). Migration and invasion are critical steps in OC growth and metastasis. Cell migration was assessed using the scratch wound healing assay. Migration in response to TGF-β1 (5 ng/mL) in OVCAR8 and CAOV3 cells was completely inhibited by exposure to IC10 of LY (Figure 2C, *p* = 0.001 for OVCAR8 and *p* < 0.001 for CAOV3 cells). Matrigel invasion assay also showed that the number of invaded cells at 24 h was significantly decreased by the IC10 of LY (Figure 2D, *p* = 0.008 for OVCAR8 and *p* = 0.009 for CAOV3 cells). These data demonstrate that LY can inhibit important phenotypic characteristics of OC cells in vitro, including proliferation, migration, and invasion. 

### 2.3. LY Inhibits TGF-β Signaling in OC Cells

**Figure 3:**
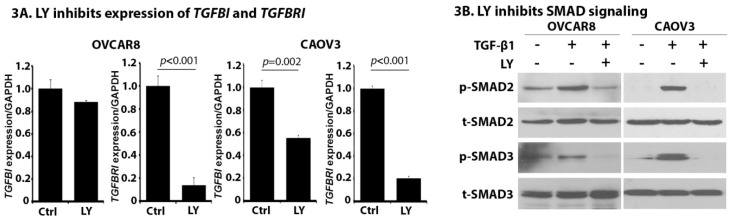
LY inhibits TGF-β signaling in OC cells. (**A**) OVCAR8 and CAOV3 cells were treated with LY for 24 h and total RNA collected for qPCR analysis. Expression of *TGFB1* and *TGFBRI* are shown. (**B**) OVCAR8 and CAOV3 cells were treated with TGF-β1 (5 ng/mL) with or without the pre-treatment of LY and total protein lysates were collected for Western blot analysis on the expression of p-SMAD2 and p-SMAD3. Total SMAD2 and SMAD3 were used as loading controls. All data represent the mean ± SD of three independent experiments.

LY functions through targeting TGFBRI and some studies have shown that LY is also able to downregulate TGFBRI expression [33,34]. The effect of LY on TGFB1 and TGFBR1 expression was assessed in HGSOC cell lines. As shown in Figure 3A, LY treatment significantly down-regulates the expression of TGFBR1 in both OVCAR8 (*p* < 0.001) and CAOV3 (*p* < 0.001) cells. We also observed a mild inhibition of TGFB1 expression in CAOV3 cells (*p* = 0.002). Consistent with these results, we observed that LY treatment inhibits TGF-β signaling characterized by the downregulation of p-SMAD2 and p-SMAD3 (Figure 3B). These data demonstrate that LY is capable of inhibiting TGF-β signaling in OC cells.

### 2.4. LY Inhibits TGF-β1-Induced Activation of NOF151-hTERT (NOF) Cells

**Figure 4:**
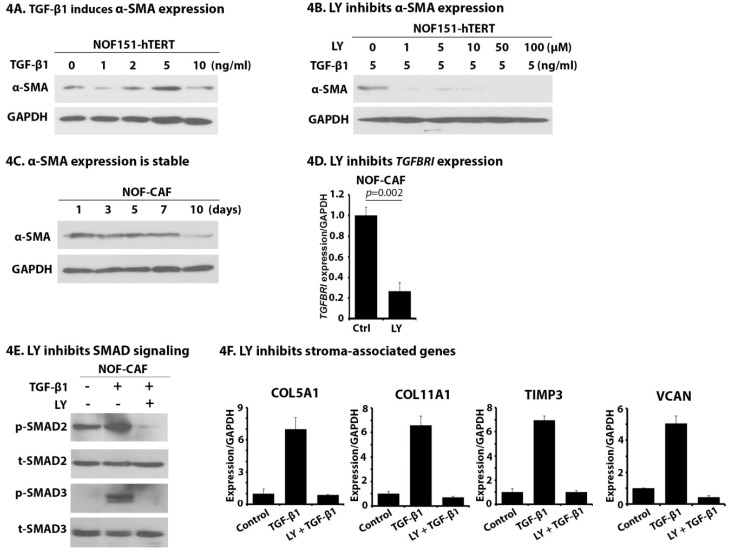
LY inhibits TGF-β1-induced activation of NOF cells, suppresses SMAD signaling and reduces the expression of HGSOC stroma-associated genes. (**A**) NOF cells were treated with increasing concentrations of TGF-β1 for 24 h and cell lysates were collected to measure the α-SMA expression by western blot. (**B**) NOF cells were pre-treated with indicated concentrations of LY for 12 h followed by the induction of TGF-β1 (5 ng/mL) for 24 h and α-SMA expression was analyzed by Western blot. (**C**) TGF-β1-activated NOF (NOF-CAF) cells were grown in TGF-β1-free Dulbecco’s modified eagle medium (DMEM). Cell lysates were collected from the 1, 3, 5, 7, and 10 days and α-SMA expression was analyzed by Western blot. (**D**) NOF-CAF cells were treated with LY (1 μM) for 24 h and TGF-βR1 expression was analyzed by qPCR. (**E**) NOF-CAF cells were treated with LY for 48 h and p-SMAD2 and p-SMAD3 expression were analyzed by Western blot. Total SMAD2 and SMAD3 were used as loading controls. (**F**) NOF-CAF cells were co-cultured with OVCAR8 cells using the Transwell system. TGF-β1 was added with or without the pre-treatment with LY and total RNA from TGF-β1-activated NOF cells collected to determine the expression of *COL5A1*, *COL11A1*, *TIMP3*, and *VCAN* by qPCR.

Cancer-associated fibroblasts (CAFs) stimulate OC growth, and TGF-β1 is capable of inducing the activation of fibroblasts [35,36,37,38]. We treated NOF fibroblast cells with increasing concentrations of TGF-β1 and observed that expression of alpha-smooth muscle actin (α-SMA), the most-widely used CAF biomarker, was gradually enhanced, with the maximal expression observed at a concentration of 5 ng/mL of TGF-β1 (Figure 4A), suggesting successful activation of NOF cells. We also observed that once activated, the expression of α-SMA was independent of TGF-β1 treatment (Figure 4C), suggesting the activation of NOF is stable. LY is known to selectively target TGFBR1 at concentrations as low as 1 μM [23]. Pretreatment of NOF cells with LY at 1 μM was able to block TGF-β1-induced α-SMA expression (Figure 4B). Similar to what we observed in OC cells (Figure 3A), LY inhibited the expression of TGFBR1 in TGF-β1-activated NOF cells (NOF-CAF) (Figure 4D, *p* = 0.002). NOF-CAF cells’ expression of p-SMAD2 and p-SMAD3 in response to TGF-β1 demonstrated intact TGF-β signaling. Importantly, when NOF-CAF cells were pretreated with LY, TGF-β1 induced p-SMAD2 and p-SMAD3 expression was abrogated (Figure 4E). These results demonstrate the LY inhibits the TGFBR1 expression and downstream TGF-β signaling in NOF-CAF cells. We measured the ability of NOF-CAF cells to express important stroma-associated genes in OC in response to TGF-β1 and the ability of LY to inhibit this expression. As shown in Figure 4F, after exposure to TGF-β1, we saw strong induction of all four tested genes, including *COL5A1*, *COL11A1*, *TIMP3*, and *VCAN*. These inductions were blocked by pretreatment with LY. Collectively, these data demonstrate that LY pretreatment suppresses the crosstalk between cancer and stroma through inhibiting the fibroblast activation and interruption of TGF-β1-induced expression of stromal genes important in OC tumors. 

### 2.5. LY Delays Tumor Growth In Vivo

To test the hypothesis that LY treatment could inhibit tumor growth and/or engraftment through targeting the tumor microenvironment, we tested the efficacy of LY in vivo. Three OC animal models were included into this study: one OC cell line (OVCAR8)-derived model (OV8-CDX) and two PDXs, PH003 and PH095. PH003 is a fast-growing PDX model derived from an ovarian carcinosarcoma that is highly resistant to traditional chemotherapy [29]. The PH095 model was derived from a HGSOC (TCGA mesenchymal subtype) patient tumor. Previous publication indicated that efficacy of LY correlated with pretreatment expression of TGFBRI mRNA and p-SMAD2 protein expression in cancer PDX models [23]. We therefore confirmed expression of TGFBRI (Figure 1E) and p-SMAD2 expression (Supplementary Figure S1) in the three models selected.

**Figure 5:**
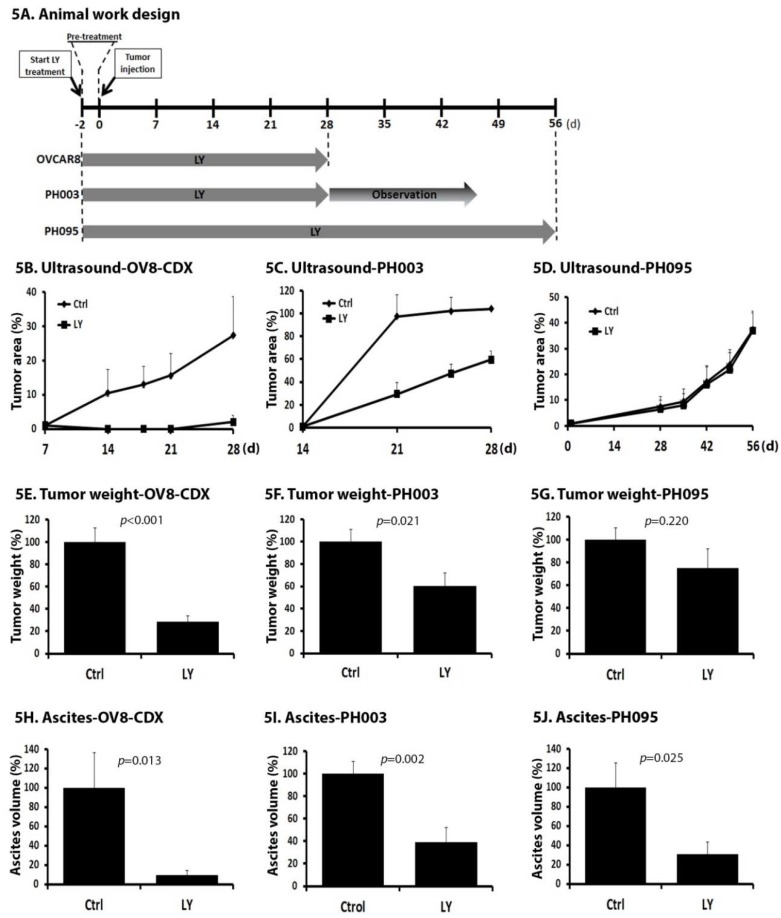
LY delays OC tumor growth and ascites development in vivo. (**A**) Scheme of animal model (cell line-derived and patient-derived xenografts) study design. LY treatment was initiated 2 days prior to tumor injection followed by continuous drug treatment. (**B**–**D**) Percentage of change in tumor area over time in three models, respectively. (**E**–**G**) Percentage of change in final tumor weights measured at harvest in three models, respectively. (**H**–**J**) Percentage of change in ascites volume measured at harvest in three models, respectively.

Mice were randomly assigned to non-treatment (control) or LY treatment group. For all models tested, mice were pre-treated for 48 h prior to intraperitoneal (IP) tumor injection and treated continuously for 4 or 8 weeks dependent upon the growth rates of the models used (Figure 5A). OV8-CDX mice were treated for 4 weeks prior to sacrifice for tumor measurement and harvested for later correlative studies. Ultrasound (US) measurement was performed to monitor tumor formation.

As shown in Figure 5B, LY treatment significantly slowed the growth of tumors in OV8-CDX. Consistent with the US results, final tumor weight was significantly reduced in mice receiving LY compared with control (*p* < 0.001, Figure 5E). Model PH003 was treated for 4 weeks, and we observed until mice met criteria for sacrifice. We observed a similar effect of LY on the inhibition of tumor growth (Figure 5C) and tumor weight (*p* = 0.021, Figure 5F) with no significant survival differences (data not shown). Because of the slower growth characteristics of model PH095 (mice typically require 3–5 weeks to form a detectable tumor after intraperitoneal (IP) injection), the treatment phase was continued for 8 weeks. LY treatment had no significant impact on the tumor growth (Figure 5D) and final tumor weight (*p* = 0.220, Figure 5G). These data demonstrate that LY can inhibit the tumor growth, or perhaps slow engraftment, in ovarian xenograft models. 

### 2.6. LY Inhibits the Development of Ascites

Reduction of ascites, even in the absence of tumor inhibition, is an important therapeutic and palliative goal. Malignant ascites is the excessive accumulation of fluid in the abdominal cavity and is commonly associated with primary and recurrent OC and is a significant cause of morbidity and mortality. Progression of ascites is associated with deterioration in the quality of life for patients [39,40]. The three models chosen for studies characteristically develop ascites. Ascites was measured using 14-mL conical tubes at the time of sacrifice. In OV8-CDX, the ascites volume was drastically reduced with LY treatment (*p* = 0.013, Figure 5H). We observed similar reduction in the volume of ascites in the two PDX models as well (*p* = 0.002 for the PH003 model, and *p* = 0.025 for the PH095 model. Figure 5I,J). These data demonstrate that LY significantly inhibits ascites formation and may be an important opportunity for palliative relief in OC.

### 2.7. LY Inhibits TGF-β Signaling and Cancer-Stroma Crosstalk

**Figure 6:**
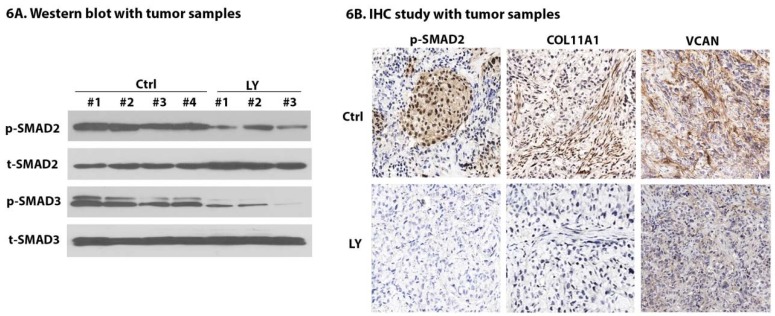
LY downregulates TGF-β signaling and expression of collagen type XI chain 1 (COL11A1) and versican VCAN in vivo. (**A**) Total protein lysates were collected from control (*n* = 4) and LY-treated mice (*n* = 3) in the OV8-CDX model. Western blot was performed to assess the expression of p-SMAD2 and p-SMAD3. Total SMAD2 and SMAD3 were used as loading controls. (**B**) IHC was performed to measure the expressions of p-SMAD2, COL11A1, and VCAN following LY treatment in OV8-CDX tumors. Magnification, 40×.

To determine if the LY effects seen in vitro (Figure 3B and Figure 4E,F) could be observed in an in vivo model, tissue samples from the OV8-CDX model were assessed to measure the effects of LY on TGF-β1-dependent signaling and expression. Specifically, our in vitro experiments (Figure 3B and Figure 4E) demonstrated that LY treatment inhibits TGF-β signaling characterized by the downregulation of p-SMAD2 and p-SMAD3. To assess this in vivo using the entire tumor specimen (cancer and stromal cells) total protein lysates were collected from 4 control mice and 3 treatment mice. Western blot analysis showed that LY treatment significantly downregulated the expression of p-SMAD2 and p-SMAD3 (Figure 6A). Immunohistochemistry (IHC) staining of p-SMAD2 also confirmed the inhibition of TGF-β signaling following LY treatment (Figure 6B). We next examined expression of COL11A1 and VCAN, which we previously demonstrated were downregulated in response to LY. We observed near-absent expression in LY treatment group mice vs. control (Figure 6B). These results indicate that LY is capable of reducing TGF-β signaling and block the cancer–stroma crosstalk in ovarian cancer-derived xenografts. 

## 3. Discussion

In this study, we demonstrate that TGF-β1 ligand and receptor are expressed in OC cells and xenograft models and are capable of downstream signaling in response to TGF-β1. Inhibition of the TGF-β signaling pathway by LY, a TGFBR1 inhibitor, reduces cell proliferation, migration, and invasion in vitro. Additionally, LY can prevent the TGF-β1-induced activation of fibroblasts and expression of important stroma-derived proteins in the cancer–stroma interplay. These results support the hypothesis that an anti-TGF-β signaling strategy may be useful in OC. Our data demonstrate that LY can delay tumor growth and ascites formation in OC xenografts and these in vivo effects are correlated with reduced-expression of important stroma-derived proteins COL11A1 and VCAN.

TGF-β signaling has important roles in tumor initiation, development, and metastasis and its deregulation is common in cancers including OC. TGF-β signaling has been considered an appropriate target for cancer therapy and several TGF-β inhibitors are in clinical investigations [14]. LY is a TGFBRI inhibitor that has been tested in many cancers, including breast and hepatocellular cancers as well as glioma, and has been shown to have efficacy when combined with standard chemotherapeutic agents. The tolerability and safety of LY have already been demonstrated in several clinical trials [27,28,41,42,43,44]. LY monotherapy has been shown to be efficacious, providing similar median overall survival (OS) as lomustine with reduced toxicity in glioblastoma. [28]. However, there is no data available in OC. LY appears to have only limited direct effect on established tumors [23,45]. However, given the crucial role of TGF-β signaling in the crosstalk between cancer and stroma, we hypothesized that LY can inhibit tumor growth and/or engraftment by decreasing the stromal receptivity. Others have demonstrated the ability of LY to inhibit metastasis in high expressing TGF-β organoids [9,24,46]. We therefore initiated treatment with LY 2 days prior to IP injection of OC cells to modulate the stroma. This “earlier and longer-time treatment” design can: (1) test our hypothesis that targeting stroma can modulate tumor development, and (2) simulate a clinical paradigm of maintenance therapy following either primary debulking surgery or induction chemotherapy (minimal residual disease models). Our results (Figure 5) support the conclusion that LY inhibits OC growth and suggest it may delay or reduce engraftment. Our experiment was not designed to specifically discern between inhibitions of growth vs. engraftment. The observation that LY treatment is associated with reduced expression of VCAN and COL11A1 in tumor stroma provides evidence of a potential TGF-β-dependent mechanism of growth inhibition. 

Another clinically relevant finding from LY monotherapy was the observation that LY inhibited ascites development. Ascites formation is responsible for significant morbidity in advanced stage OC and is a major contributor to poor quality of life as it is often associated with loss of gastrointestinal function and mobility. Current palliative options are limited and include serial drainage and indwelling invasive catheters for prolonged drainage—both of which offer only short-term relief and carry risks. Targeted therapies, including the anti-angiogenic targeted agent bevacizumab, the anti-vascular endothelial growth factor trap (VEGF-Trap) aflibercept, and a tri-functional monoclonal antibody have recently been developed as novel therapeutic options for malignant ascites [47]. Our study shows that LY can inhibit ascites development even in models where we did not observe a reduction in tumor growth (Figure 5D,G,J). This finding indicates that anti-TGF-β signaling may offer palliation for OC patients with malignant ascites. 

Our study has some limitations. We used TGF-β1-induced fibroblasts to study the impact of LY on CAFs. We feel this is justified given that TGF-β1 is capable of inducing fibroblast activation [35,36,37,38] and that TGF-β1-activated fibroblasts share features with CAFs in transcriptome profiling, metabolic programing, and tumor stimulation in cancer [35,36,38]. Use of isolated CAFs from tumor may be preferable; however, CAFs have heterogeneous origins, phenotype, and functions which can also lead to erroneous biological responses. Yeung et al. identified two distinct subtypes of CAFs in OC: the CAF-C subtype correlated with poor survival, and CAF-N subtype did not [35]. Use of TGF-β1-activated fibroblasts overcomes the limitation of CAFs in heterogeneity; nevertheless, this is a very simplified model which is not able to fully reproduce the effect of endogenous CAFs. Further study of the interplay between cancer cells and CAFs, by using CAFs cells isolated from patient tumor would be important next steps. Additionally, the mouse strain used in our studies, NSG, is immune-deficient, lacking mature T cells, B cells, and NK cells. TGF-β signaling has been shown to help tumor cells escape from the immune-surveillance through targeting both T cells and NK cells within the tumor microenvironment [48,49,50]. Use of an immune-deficient mouse strain limits our ability to study the effect of LY on immune function, which may be quite important [51]. However, we would anticipate a stronger anticancer effect in immune competent models [26,50]. There is also a need for reliable biomarkers of response. The identification of such biomarkers allows the identification of the subgroup of patients most likely to derive benefit from therapy. In ovarian cancer one such example is the discovery that defects in homologous recombination sensitize cells to poly (ADP-ribose) polymerase (PARP) inhibitor therapy. This maximizes benefits while reducing harm or the use of testing and using ineffective therapy in other patients. We used the expression of TGFBRI mRNA and p-SMAD2 protein as criteria to select models to study LY efficacy, and using these criteria observed two out of three models (OV8-CDX, PH003) responded well to LY. Not surprisingly, this implies multiple other factors are important in whether TGF-β signaling inhibition will be effective. Yuan et al. identified down-regulation of SKI like proto-oncogene (SKIL) and prostate transmembrane androgen-induced 1 protein (PMEPA1) after LY treatment as biomarkers for response in hepatocellular carcinoma (HCC) patients [52]. We did not see the inhibition of these two genes in the tumor samples from our LY-treatment group (data not shown). The identification of reliable predictive biomarkers to identify patients who are most likely to benefit from TGF-β inhibitor treatment should be a goal of future clinical trials [26].

## 4. Materials and Methods

### 4.1. Cell Culture and Reagents

All OC cell lines were maintained in Dulbecco’s modified eagle medium (DMEM) medium (Corning) with 10% fetal bovine serum (FBS), 100 units/mL penicillin, and 100 μg/mL of streptomycin in humidified air with 5% CO_2_ at 37 °C. The NOF151-hTERT (NOF) cell is an immortalized normal fibroblast cell line, kindly provided by Professor Jinsong Liu (MD Anderson, TX, USA). NOF cells were maintained in MCDB 105 and 199 media (Sigma-Aldrich, St. Louis, MO, USA) supplemented with 15% FBS (ThermoFisher, Waltham, MA, USA) and antibiotics [53]. LY was kindly provided by Eli Lilly (Indianapolis, IN, USA). For in vitro study, LY was dissolved in DMSO at 10 mM and kept at −20 °C. For in vivo experiment, LY was freshly formulated on the night before the treatment in 1% carboxymethylcellulose/0.5% sodium lauryl sulfate/0.085% povidone.

### 4.2. Cell Proliferation and Clonogenic Assay

To evaluate the effect of LY on cell viability, OVCAR8 and CAOV3 cells were plated in 96-well plates at 3000 cells/well followed by treatment with increasing concentration of LY (0, 0.1, 0.2, 0.6, 1.0, 1.5 mM) for 72 h. CellTiter 96^®^ AQeous One Solution (Promega, Fitchburg, WI, USA) was used to quantify the cell proliferation according to the manufacturer’s manual. For clonogenic assay, cells were plated in 6-well plates at 1000 cells/well followed by treatment with IC25 concentration of LY for 24 h. Media was replaced with normal medium and cells allowed to grow for 15 days. Colonies were fixed with methanol and stained with crystal violet. The stained colonies were counted under bright-field microscopy and data were analyzed by using GraphPad Prism 7 (GraphPad Software, San Diego, CA, USA). 

### 4.3. Wound Healing Scratch Assay

OVCAR8 and CAOV3 cells were cultured in 6-well plates. Following serum starvation for overnight, the 90% confluent cells were scratched with a 1-mL pipette tip. TGF-β1 (R&D System, dissolved at 5 ng/mL) was added into the medium with or without the pretreatment of LY. Photographs of the same area of the wound were taken after 24 h of the scratch. The width of the wound was measured with Image J software (National Institutes of Health, Bethesda, MD, USA) and the rate of wound closure was calculated. The experiment was repeated three times. 

### 4.4. Matrigel Cell Invasion Assay

Cell invasion was examined using 24-well Matrigel invasion chambers (Corning, Corning, NY, USA) with an 8-µm pore membrane. Briefly 50,000 cells were serum-starved for overnight and plated into serum-free medium onto Matrigel-coated upper chamber. Five hundred microliters of serum-free medium containing 5 ng/mL of TGF-β1 with or without pretreatment of LY were added to the bottom well. After 24 h, the upper surface of the insert was wiped gently with cotton swabs to remove non-migrating cells. Cells that had migrated to the lower surface were stained and counted. The experiment was repeated three times. 

### 4.5. Microarray and Real-Time Quantitative PCR (qPCR)

Total RNA was isolated from cells or tissues by using RNeasy Mini Kit (Qiagen, Hilden, Germany). Equal amounts of RNA were transcribed to cDNA with SuperScript II reverse transcriptase using random primers (ThermoFisher, Waltham, MA, USA). Microarray analysis was performed as previously described [29]. Real-time qPCR was performed by using Power SYBR^®^ Green Master Mix (ThermoFisher, Waltham, MA, USA). Glyceraldehyde 3-phosphate dehydrogenase (GAPDH) was used as an internal control.

### 4.6. Western Blotting Analysis

Cells were collected and lysed in radioimmunoprecipitation assay (RIPA) buffer supplemented with a protease inhibitor cocktail (Sigma). Equal amounts of protein were analyzed on SDS-PAGE gels. Primary antibodies were purchased from Sigma (p-SMAD2, GAPDH), Abcam (p-SMAD3), and Thermo (COL11A1).

### 4.7. OC Animal Models

The animal protocol was reviewed and approved by the Institutional Animal Care and Use Committee (IACUC) of Mayo Clinic on 06/03/2015 (IACUC Protocol A25015-15). The in vivo experiments utilized 6–8 weeks-old female NOD.CB17/Prkdcscid/NCrHsd (NSG) mice (Jackson Laboratory, Bar Harbor, ME, USA), which were housed in a pathogen-free environment. Mice were implanted using our previously described intraperitoneal xenograft model of OC [29]. Briefly, 1 × 10^6^ of OVCAR8 cells were used to generate cell line-derived xenografts (OV8-CDX) and 0.1 g of low-passaged PDX tumors were used to generate the two PDXs (PH003, PH095, Supplementary Table S1). OV8-CDX, PH003-PDX, and PH095-PDX models (*n* = 5–10 mice/arm) were treated with LY (75 mg/kg, twice-daily, 7 days a week) and compared to non-treatment controls. In all models, mice were pretreated for 2 days before xenografting, then treated for 4 weeks (OV8-CDX and PH003) or 8 weeks (PH095). Ultrasound (US) measurement was used to monitor tumor growth [29]. At day 28, we (1) collected tumor (OV8-CDX); or (2) observed until progression (PH003). At day 56, we collected tumor on PH095 model. OV8-CDX tumors were used for target gene analysis.

### 4.8. Tissue Processing and Immunohistochemistry (IHC)

Tissue collected from mice were fixed in buffered formalin (Fisher Scientific, Hampton, NH, USA) and processed in the tissue core facility at Mayo Clinic, AZ. IHC staining was carried out using 5-µm tissue sections according to standard procedures. Briefly, after antigen retrieval with EDTA buffer (pH 8.0, Newcomer Supply, Middleton, WI, USA), samples were blocked and followed by primary antibody incubation at 4 °C for overnight. A secondary antibody (Cell signaling, Danvers, MA, USA) was applied for 30 min at room temperature. Chromogenic detection of protein expression was determined in the presence of diaminobenzidine (DAB, Agilent, Santa Clara, CA, USA) and scanned by a Desktop scanner (Objective Imaging, Kansasville, WI, USA). Primary antibodies purchased from Thermo (p-SMAD2, COL11A1) and Abcam (VCAN).

### 4.9. Statistical Analysis

All values are shown as the mean ± standard deviation (SD) of three individual experiments performed in triplicate and presented as the mean. Statistical analysis was performed using Student’s *t*-test with GraphPad Prism 7 statistical software. The significance of difference was shown by the *p* value (* *p* < 0.05, ** *p* < 0.01). 

## 5. Conclusions 

In summary, our study is the first to show that LY alone is able to inhibit tumor growth and ascites formation in OC xenografts. The progression-free survival (PFS) of patients with OC after first-line platinum-based therapy has remained remarkably constant over the last two decades, suggesting a need for maintenance therapy to extend remissions. Our results support the efficacy of LY in modulating the tumor microenvironment which impedes growth and/or engraftment of OC. These findings provide preclinical support for testing LY as maintenance therapy in high-risk OC clinical trials since the safety of LY in patient has been proven, as well as for future studies to determine potential synergy with existing therapies [33,43,54].

## Supplementary Materials

The following are available online at http://www.mdpi.com/2072-6694/10/8/260/s1, Figure S1: Expression of phosphorylated-SMAD2 across animal models. Protein lysates were extracted from untreated OV8-CDX, PH003, and PH095 models and western blot were performed to assess the expression of p-SMAD2. Total SMAD2 was used as loading control; Table S1: PDX models minimal information standard.

## References

[1] Torre L.A., Bray F., Siegel R.L., Ferlay J., Lortet-Tieulent J., Jemal A. (2015). Global cancer statistics, 2012. CA Cancer J. Clin..

[2] Siegel R.L., Miller K.D., Jemal A. (2018). Cancer statistics, 2018. CA Cancer J. Clin..

[3] Schorge J.O., McCann C., Del Carmen M.G. (2010). Surgical debulking of ovarian cancer: What difference does it make?. Rev. Obstet. Gynecol..

[4] Nick A.M., Coleman R.L., Ramirez P.T., Sood A.K. (2015). A framework for a personalized surgical approach to ovarian cancer. Nat. Rev. Clin. Oncol..

[5] Bast R.C. (2011). Molecular approaches to personalizing management of ovarian cancer. Ann. Oncol..

[6] Khalique S., Hook J.M., Ledermann J.A. (2014). Maintenance therapy in ovarian cancer. Curr. Opin. Oncol..

[7] Burger R.A., Brady M.F., Bookman M.A., Fleming G.F., Monk B.J., Huang H., Mannel R.S., Homesley H.D., Fowler J., Greer B.E. (2011). Incorporation of bevacizumab in the primary treatment of ovarian cancer. N. Engl. J. Med..

[8] Perren T.J., Swart A.M., Pfisterer J., Ledermann J.A., Pujade-Lauraine E., Kristensen G., Carey M.S., Beale P., Cervantes A., Kurzeder C. (2011). A phase 3 trial of bevacizumab in ovarian cancer. N. Engl. J. Med..

[9] Yeung T.L., Leung C.S., Wong K.K., Samimi G., Thompson M.S., Liu J., Zaid T.M., Ghosh S., Birrer M.J., Mok S.C. (2013). TGF-β modulates ovarian cancer invasion by upregulating CAF-derived versican in the tumor microenvironment. Cancer Res..

[10] Cheon D.J., Tong Y., Sim M.S., Dering J., Berel D., Cui X., Lester J., Beach J.A., Tighiouart M., Walts A.E. (2014). A collagen-remodeling gene signature regulated by TGF-β signaling is associated with metastasis and poor survival in serous ovarian cancer. Clin. Cancer Res..

[11] Neuzillet C., Tijeras-Raballand A., Cohen R., Cros J., Faivre S., Raymond E., de Gramont A. (2015). Targeting the tgfbeta pathway for cancer therapy. Pharmacol. Ther..

[12] Akhurst R.J., Hata A. (2012). Targeting the tgfbeta signalling pathway in disease. Nat. Rev. Drug. Discov..

[13] Pickup M., Novitskiy S., Moses H.L. (2013). The roles of tgfbeta in the tumour microenvironment. Nat. Rev. Cancer.

[14] Sheen Y.Y., Kim M.J., Park S.A., Park S.Y., Nam J.S. (2013). Targeting the transforming growth factor-β signaling in cancer therapy. Biomol. Ther..

[15] Thomas D.A., Massague J. (2005). TGF-β directly targets cytotoxic T cell functions during tumor evasion of immune surveillance. Cancer Cell.

[16] Yang L., Pang Y., Moses H.L. (2010). TGF-β and immune cells: An important regulatory axis in the tumor microenvironment and progression. Trends. Immunol..

[17] Viel S., Marcais A., Guimaraes F.S., Loftus R., Rabilloud J., Grau M., Degouve S., Djebali S., Sanlaville A., Charrier E. (2016). TGF-β inhibits the activation and functions of NK cells by repressing the mtor pathway. Sci. Signal..

[18] Jia D., Liu Z., Deng N., Tan T.Z., Huang R.Y., Taylor-Harding B., Cheon D.J., Lawrenson K., Wiedemeyer W.R., Walts A.E. (2016). A COL11A1-correlated pan-cancer gene signature of activated fibroblasts for the prioritization of therapeutic targets. Cancer Lett..

[19] Bhola N.E., Balko J.M., Dugger T.C., Kuba M.G., Sanchez V., Sanders M., Stanford J., Cook R.S., Arteaga C.L. (2013). TGF-β inhibition enhances chemotherapy action against triple-negative breast cancer. J. Clin. Investig..

[20] Bueno L., de Alwis D.P., Pitou C., Yingling J., Lahn M., Glatt S., Troconiz I.F. (2008). Semi-mechanistic modelling of the tumour growth inhibitory effects of LY2157299, a new type I receptor TGF-β kinase antagonist, in mice. Eur. J. Cancer.

[21] Dituri F., Mazzocca A., Fernando J., Papappicco P., Fabregat I., De Santis F., Paradiso A., Sabba C., Giannelli G. (2013). Differential inhibition of the TGF-β signaling pathway in HCC cells using the small molecule inhibitor LY2157299 and the D10 monoclonal antibody against TGF-β receptor type II. PLoS ONE.

[22] Giannelli G., Villa E., Lahn M. (2014). Transforming growth factor-β as a therapeutic target in hepatocellular carcinoma. Cancer Res..

[23] Maier A., Peille A.L., Vuaroqueaux V., Lahn M. (2015). Anti-tumor activity of the TGF-β receptor kinase inhibitor galunisertib (LY2157299 monohydrate) in patient-derived tumor xenografts. Cell Oncol..

[24] Calon A., Lonardo E., Berenguer-Llergo A., Espinet E., Hernando-Momblona X., Iglesias M., Sevillano M., Palomo-Ponce S., Tauriello D.V., Byrom D. (2015). Stromal gene expression defines poor-prognosis subtypes in colorectal cancer. Nat. Genet..

[25] Alsina-Sanchis E., Figueras A., Lahiguera A., Vidal A., Casanovas O., Graupera M., Villanueva A., Vinals F. (2016). The TGF-β pathway stimulates ovarian cancer cell proliferation by increasing IGF1R levels. Int. J. Cancer.

[26] De Gramont A., Faivre S., Raymond E. (2017). Novel TGF-β inhibitors ready for prime time in onco-immunology. Oncoimmunology.

[27] Fujiwara Y., Nokihara H., Yamada Y., Yamamoto N., Sunami K., Utsumi H., Asou H., Takahash I.O., Ogasawara K., Gueorguieva I. (2015). Phase 1 study of galunisertib, a TGF-β receptor I kinase inhibitor, in Japanese patients with advanced solid tumors. Cancer Chemother. Pharmacol..

[28] Brandes A.A., Carpentier A.F., Kesari S., Sepulveda-Sanchez J.M., Wheeler H.R., Chinot O., Cher L., Steinbach J.P., Capper D., Specenier P. (2016). A phase II randomized study of galunisertib monotherapy or galunisertib plus lomustine compared with lomustine monotherapy in patients with recurrent glioblastoma. Neuro. Oncol..

[29] Weroha S.J., Becker M.A., Enderica-Gonzalez S., Harrington S.C., Oberg A.L., Maurer M.J., Perkins S.E., AlHilli M., Butler K.A., McKinstry S. (2014). Tumorgrafts as in vivo surrogates for women with ovarian cancer. Clin. Cancer Res..

[30] Gore J., Imasuen-Williams I.E., Conteh A.M., Craven K.E., Cheng M., Korc M. (2016). Combined targeting of TGF-β, EGFR and HER2 suppresses lymphangiogenesis and metastasis in a pancreatic cancer model. Cancer Lett..

[31] Zhang C., Chen W., Zhang X., Huang B., Chen A., He Y., Wang J., Li X. (2016). Galunisertib inhibits glioma vasculogenic mimicry formation induced by astrocytes. Sci. Rep..

[32] Domcke S., Sinha R., Levine D.A., Sander C., Schultz N. (2013). Evaluating cell lines as tumour models by comparison of genomic profiles. Nat. Commun..

[33] Herbertz S., Sawyer J.S., Stauber A.J., Gueorguieva I., Driscoll K.E., Estrem S.T., Cleverly A.L., Desaiah D., Guba S.C., Benhadji K.A. (2015). Clinical development of galunisertib (LY2157299 monohydrate), a small molecule inhibitor of transforming growth factor-β signaling pathway. Drug Des. Dev. Ther..

[34] Liu X., Yu M., Chen Y., Zhang J. (2016). Galunisertib (LY2157299), a transforming growth factor-β receptor I kinase inhibitor, attenuates acute pancreatitis in rats. Braz. J. Med. Biol. Res..

[35] Yeung T.L., Sheng J., Leung C.S., Li F., Kim J., Ho S.Y., Matzuk M.M., Lu K.H., Wong S.T.C., Mok S.C. (2018). Systematic identification of druggable epithelial-stromal crosstalk signaling networks in ovarian cancer. J. Natl. Cancer Inst..

[36] Zhang D., Wang Y., Shi Z., Liu J., Sun P., Hou X., Zhang J., Zhao S., Zhou B.P., Mi J. (2015). Metabolic reprogramming of cancer-associated fibroblasts by IDH3α downregulation. Cell Rep..

[37] Li Q., Zhang D., Wang Y., Sun P., Hou X., Larner J., Xiong W., Mi J. (2013). MiR-21/Smad 7 signaling determines TGF-β1-induced CAF formation. Sci. Rep..

[38] Guido C., Whitaker-Menezes D., Capparelli C., Balliet R., Lin Z., Pestell R.G., Howell A., Aquila S., Ando S., Martinez-Outschoorn U. (2012). Metabolic reprogramming of cancer-associated fibroblasts by TGF-β drives tumor growth: Connecting TGF-β signaling with “Warburg-like” cancer metabolism and L-lactate production. Cell Cycle.

[39] Ayantunde A.A., Parsons S.L. (2007). Pattern and prognostic factors in patients with malignant ascites: A retrospective study. Ann. Oncol..

[40] Kipps E., Tan D.S., Kaye S.B. (2013). Meeting the challenge of ascites in ovarian cancer: New avenues for therapy and research. Nat. Rev. Cancer.

[41] Ikeda M., Takahashi H., Kondo S., Lahn M.M.F., Ogasawara K., Benhadji K.A., Fujii H., Ueno H. (2017). Phase 1b study of galunisertib in combination with gemcitabine in japanese patients with metastatic or locally advanced pancreatic cancer. Cancer Chemother. Pharmacol..

[42] Rodon J., Carducci M., Sepulveda-Sanchez J.M., Azaro A., Calvo E., Seoane J., Brana I., Sicart E., Gueorguieva I., Cleverly A. (2015). Pharmacokinetic, pharmacodynamic and biomarker evaluation of transforming growth factor-β receptor I kinase inhibitor, galunisertib, in phase 1 study in patients with advanced cancer. Investig. New Drugs.

[43] Kovacs R.J., Maldonado G., Azaro A., Fernandez M.S., Romero F.L., Sepulveda-Sanchez J.M., Corretti M., Carducci M., Dolan M., Gueorguieva I. (2015). Cardiac safety of TGF-β receptor I kinase inhibitor ly2157299 monohydrate in cancer patients in a first-in-human dose study. Cardiovasc. Toxicol..

[44] Rodon J., Carducci M.A., Sepulveda-Sanchez J.M., Azaro A., Calvo E., Seoane J., Brana I., Sicart E., Gueorguieva I., Cleverly A.L. (2015). First-in-human dose study of the novel transforming growth factor-β receptor I kinase inhibitor LY2157299 monohydrate in patients with advanced cancer and glioma. Clin. Cancer Res..

[45] Yeo S.K., Wen J., Chen S., Guan J.L. (2016). Autophagy differentially regulates distinct breast cancer stem-like cells in murine models via EGFR/stat3 and TGFB/SMAD signaling. Cancer Res..

[46] Bierie B., Moses H.L. (2006). Tumour microenvironment: TGF-β: The molecular Jekyll and Hyde of cancer. Nat. Rev. Cancer.

[47] Smolle E., Taucher V., Haybaeck J. (2014). Malignant ascites in ovarian cancer and the role of targeted therapeutics. Anticancer Res..

[48] Yoon J.H., Jung S.M., Park S.H., Kato M., Yamashita T., Lee I.K., Sudo K., Nakae S., Han J.S., Kim O.H. (2013). Activin receptor-like kinase5 inhibition suppresses mouse melanoma by ubiquitin degradation of smad4, thereby derepressing eomesodermin in cytotoxic t lymphocytes. EMBO Mol. Med..

[49] Dutta A., Banerjee A., Saikia N., Phookan J., Baruah M.N., Baruah S. (2015). Negative regulation of natural killer cell in tumor tissue and peripheral blood of oral squamous cell carcinoma. Cytokine.

[50] Tran H.C., Wan Z., Sheard M.A., Sun J., Jackson J.R., Malvar J., Xu Y., Wang L., Sposto R., Kim E.S. (2017). TGFβR1 blockade with galunisertib (LY2157299) enhances anti-neuroblastoma activity of the anti-GD2 antibody dinutuximab (ch14.18) with natural killer cells. Clin. Cancer Res..

[51] Ishikawa F., Yasukawa M., Lyons B., Yoshida S., Miyamoto T., Yoshimoto G., Watanabe T., Akashi K., Shultz L.D., Harada M. (2005). Development of functional human blood and immune systems in NOD/SCID/IL2 receptor {gamma} chain(null) mice. Blood.

[52] Cao Y., Agarwal R., Dituri F., Lupo L., Trerotoli P., Mancarella S., Winter P., Giannelli G. (2017). Ngs-based transcriptome profiling reveals biomarkers for companion diagnostics of the TGF-β receptor blocker galunisertib in HCC. Cell Death Dis..

[53] Yang G., Rosen D.G., Zhang Z., Bast R.C., Mills G.B., Colacino J.A., Mercado-Uribe I., Liu J. (2006). The chemokine growth-regulated oncogene 1 (GRO-1) links RAS signaling to the senescence of stromal fibroblasts and ovarian tumorigenesis. Proc. Natl. Acad. Sci. USA.

[54] Valcarcel D., Verma A., Platzbecker U., Santini V., Giagounidis A., Díez-Campelo M., Janssen J., Schlenk R.F., Gaidano G., de Oteyza J.P. (2015). Phase 2 study of monotherapy galunisertib (LY2157299 monohydrate) in very low-, low-, and intermediate-risk patients with myelodysplastic syndromes. Blood.

