# Supplementary material for: LY2157299 Monohydrate, a TGF-βR1 Inhibitor, Suppresses Tumor Growth and Ascites Development in Ovarian Cancer
Source: Cancers (Basel). 2018 Aug 7;10(8):260. doi: 10.3390/cancers10080260 (PMC6115954; doi:10.3390/cancers10080260)
Supplement: Supplementary file 1 [file cancers-10-00260-s001.pdf]

Supplementary Materials: LY2157299 Monohydrate, a TGF-βR1 Inhibitor, Suppresses Tumor Growth and Ascites Development in Ovarian Cancer

Qing Zhang, Xiaonan Hou, Bradley J. Evans, Jamison L. VanBlaricom, Saravut J. Weroha and William A. Cliby

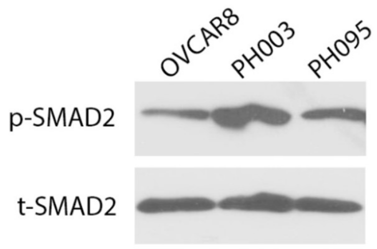

Figure S1. Expression of phosphorylated-SMAD2 across animal models. Protein lysates were extracted from untreated OV8-CDX, PH003, and PH095 models and western blot were performed to assess the expression of p-SMAD2. Total SMAD2 was used as loading control.

Table S1. PDX models minimal information standard

Module	Field	Value (PH003)	Value (PH095)
	Patient ID	3	95
	Gender	Female	Female
Clinical/Patient	Age	59	65
	Diagnosis	Ovarian Cancer	Ovarian Cancer
	Consent to share data	Academic centers only	Academic centers only
	Tumor ID	PH003	PH095
	Primary Tissue of Origin	Ovary	Ovary
	Collection Site	Primary	Primary
	Specimen tumor tissue	Ovary	Ovary
Clinical/Tumor	Histology	Carcinosarcoma	Serous
	Grade	High	High
	Stage	IIIC	IV
	Markers	N/A	N/A
	Treatment status	Naïve	Naïve
	PDX ID	PH003	PH095
	Mouse strain	NOD.CB17/Prkdcscid/NCrHsd (NSG)	NOD.CB17/Prkdcscid/NCrHsd (NSG)
	Strain immune system humanized	No	No
	Tumor preparation	Solid tumor	Solid tumor
Model creation	Injection type and site	Intraperitoneal	Intraperitoneal
	Tumor characterization technology	Histology and IHC	Histology and IHC
	Tumor confirmed not to be of mouse/EBV origin	Yes, negative for CD45	Yes, negative for CD45
	Passage QA performed	Passage P3	Passage P5

© 2018 by the authors. Licensee MDPI, Basel, Switzerland. This article is an open access article distributed under the terms and conditions of the Creative Commons Attribution (CC BY) license (http://creativecommons.org/licenses/by/4.0/).
